# The utilization of traditional, complementary and alternative medicine for non-communicable diseases and mental disorders in health care patients in Cambodia, Thailand and Vietnam

**DOI:** 10.1186/s12906-016-1078-0

**Published:** 2016-03-08

**Authors:** Karl Peltzer, Supa Pengpid, Apa Puckpinyo, Siyan Yi, Le Vu Anh

**Affiliations:** ASEAN Institute for Health Development, Mahidol University, Salaya, Thailand; Department of Psychology, University of the Free State, Bloemfontein, South Africa; HIV/STIs and TB Research Programme, Human Sciences Research Council, Pretoria, South Africa; Department of Research and Innovation, University of Limpopo, Turfloop, South Africa; KHANA, Phnom Penh, Cambodia; Vietnam Public Health Association, Ha Noi, Vietnam

**Keywords:** Utilization, Traditional, Complementary medicine, Chronic disease patients, Cambodia, Thailand, Vietnam

## Abstract

**Background:**

The purpose of our study was to determine the prevalence of traditional, complementary and alternative medicine (TCAM) use in patients with chronic diseases in lower Mekong countries.

**Methods:**

A cross-sectional study was conducted in a health care setting using a random sample of 4799 adult patients (Mean age: 52.3 years, SD = 22.7) with chronic diseases in Cambodia, Vietnam and Thailand. The measure included the International Questionnaire to measure usage of complementary and alternative medicine (I-CAM).

**Results:**

The 1 year prevalence of consulting TCAM providers was 26.0 %; 27.0 % in Cambodia, 26.3 % in Thailand, 23.9 % in Vietnam. The most commonly consulted TCAM providers were the herbalist (17.3 %), massage therapist (6.0 %), and acupuncturist (5.5 %). For all different types of TCAM providers more than 80 % of participants perceived the consultation as very or somewhat helpful. The own use of herbal medicine was 41.0 %, own use of vitamins 26.5 % and the own use of other supplements 9.7 % in the past 12 months. The most common self-help practices in the past 12 months included praying for your own health (30.1 %), meditation (13.9 %) and relaxation techniques (9.9 %). In multivariate logistic regression analyses, older age, rural residence and having two or more chronic conditions was associated with the use a TCAM provider; being female, urban residence, residing in Vietnam and having two or more chronic conditions was associated with the use of TCAM products; and being female, older age, rural residence, higher formal education, and residing in Cambodia was associated with the use of TCAM self-help practices.

**Conclusions:**

TCAM use is common among chronic disease patients in lower Mekong countries and is associated with several sociodemographic and disease specific factors.

## Background

Large populations in Asian countries utilize traditional medicine [1]. The World Health Organization [2] emphasised the importance of the study on the prevalence and determinants of Traditional, Complementary and Alternative Medicine (TCAM) use. Studies on the utilization of TCAM have been focusing on high income countries, with a use of any CAM between 9.8 to 76 % [3]. Although many populations in lower Mekong and Association of Southeast Asian Nations (ASEAN) are reported to use TCAM to help meet their health care needs, precise data are lacking [2].

In Cambodia, several sources [4–6] describe the estimated use of traditional medicine in Cambodia as strong and widespread. There are several types of TCAM providers in Cambodia, including the “Kru Khmer, mediums known as *Kru Chol Ruup* and Buddhist monks” [5]. TCAM in Cambodia includes three explanatory models of disease: supernatural theory, naturalistic theory, and maintenance of hot/cold balance [4]. TCAM providers, mostly operate from their homes or religious institutions in the private sector; traditional medicine is not yet integrated with conventional medicine in Cambodia [5]. Non-prescription herbal preparations are cheaply available in markets [4]. TCAM is officially supported by the Cambodian government, in particular to support primary health care [5]. There have been reports on overall low utilization of public health facilities (0.6 visits per person per year) in Cambodia, mainly due to under-resourced publicly funded health services, which leaves many people to use the private sector for treatment, particularly private pharmacies [7].

In Thailand, it is estimated that among Thai people seeking care in public health facilities 10 % receive Thai traditional medicine, which may include Thai traditional massage, herbal steam bath, traditional herbal medicines and acupuncture [8]. Traditional healers, including spiritual, herbal, and massage healers and traditional midwives, are distributed all over Thailand operating from their homes, religious institutions and health care facilities [9]. According to Thai traditional medicine, explanatory models for human illness may include supernatural powers, imbalance in the four elements of the body such as imbalance of heat and cold, positive and negative influences from the universe [10]. Thailand introduced universal health coverage from 2002. The rate of rate of out-patient health care utilization for the whole country was 3.4 visits per person per year in 2009 [11]. However, there were differences by geographic region, with higher utilization rates in provinces that are the centres of regions and the provinces in the Central region and lower utilization rates in most provinces in the Northeast of Thailand [11].

In Vietnam, traditional medicine comprises of Vietnamese traditional medicine (influenced by Chinese traditional medicine) and oriental medicine and they form an integral part of the national health care system in Vietnam providing about 30 % of health care [12]. Treatment is provided by “traditional medicine practitioners (who have not received any formal education) and by traditional medical doctors (who have graduated from a department of traditional medicine at a medical university)” [12]. In Vietnam, almost all of the TCAM components such as herbal medicine, acupuncture, acupressure, and massage are covered by the national health insurance [13]. Explanatory models for illness include spiritual causes, imbalance of hot or cold, and western concepts of disease (i.e. germ theory) [14]. The rate of out-patient health service utilization was 1.38 visits per person in 2009 [15], which was higher among health insurance enrolees (health insurance coverage was 60 % in 2010) and among the people living in urban areas [15].

Some research seems to indicate that TCAM users were more likely to suffer from one or more chronic conditions, especially mental, musculoskeletal and metabolic disorders [16–21]. In a few studies among the lower Mekong countries, mainly in Thailand, a high prevalence of TCAM use of patients in conventional health facilities for various chronic conditions such as cancer, diabetes, hypertension, asthma, and mental disorders were found [19]. For example, the prevalence of TCAM use was more than 60 % among cancer patients in Thailand [22, 23], diabetic patients 47.8 % in Thailand [24], chronic kidney disease patients 45 [25] and 76.9 % for patients with mental disorder (schizophrenia) in Cambodia [26]. Lee et al. [27] found among chronic disease patients in Singapore’s public health services polyclinics an estimated TCAM use prevalence of 22.7 % in the past year. Hasan et al. [28] found 63.9 % TCAM utilization in the past year among chronic disease patients in Malaysia. Wazaify et al. [29] reported among chronic disease patients in Jordan that 20.4 % used Hijama (bloodletting), 11.6 % other TCAM types and 7.6 % herbs, and Jiaranaikajorn and Panthawangkul [30] found that 52.5 % of medical patients in a tertiary hospital in Thailand utilized TCAM.

For the different chronic conditions in ASEAN countries a range of TCAM types has been used, ranging from herbal treatment, Chinese traditional medicine, spiritual treatment, dietary supplements, acupuncture, yoga, homoeopathy, reflexology and massage [19]. Factors associated with TCAM use in chronic disease patients include female gender [31, 32], low income [31], and high levels of education [31, 32], older age [28], specific chronic conditions, triad (stroke, arthritis or musculoskeletal) [27], mental disorder [17], cancer [17] two or more chronic diseases [32, 33], and adherence to traditional health beliefs [28].

The aim of this study was to conduct cross-sectional surveys on the prevalence and associated factors of TCAM use of chronic disease and mental disorder patients in health care settings in lower Mekong countries (Cambodia, Thailand, and Vietnam).

## Methods

### Design

In each lower Mekong country (Cambodia, Thailand, Vietnam) a cross-sectional survey in rural and urban health facilities were conducted with out-patients with chronic diseases and mental disorders.

### Sample and procedure

The sample size included at least 800 persons from four rural communities (districts) and 800 individuals from four urban communities (districts), 1600 per country and 4800 in total across all three countries. Urban or rural designation was based on the area in which the health facilities were located, i.e. metropolitan areas (urban) and non-metropolitan areas (rural). The health facility in an urban area and in a rural area was conveniently selected. In Cambodia, Chbar Ampov and Boeng Kok Health Center located in Phnom Penh and Kampong Cham provincial town were selected as urban primary healthcare settings. Skun health center in Kampong Cham and Pouk Health Center in Siem Reap were selected as rural health facilities. In Thailand, district hospitals in Songkla (*N* = 395), Suphan Buri (*N* = 100), Surin (*N* = 201), Khon Kaen (*N* = 200), Chiang Rai (*N* = 400), Samutsongkram (*N* = 200) and one tertiary hospital in Kanchanaburi (*N* = 100) across the whole country were included. In Vietnam, the data was collected in 20 health facilities (13 urban and 7 rural areas) in 11 districts of 3 Northern provinces.

Every eligible patient was selected at the health facility, using a systematic sampling procedure. We recruited all patients who access services of the selected health facilities for their treatment with some inclusion criteria including adult patients with minimum age of 21 years and who have been treated in the past 12 months for any of the 20 chronic conditions such as asthma, chronic obstructive pulmonary disease (COPD), diabetes mellitus, hypertension, dyslipidaemia, coronary artery disease, cardiac failure, cardiac arrhythmias, stroke, arthritis, cancer, gout and other musculoskeletal conditions such as chronic backache, epilepsy, Parkinson disease, liver disease, kidney disease, thyroid disease, stomach and intestinal diseases, and mental disorders [27, 34]. The health facility staff conducted the screening for these two inclusion criteria and referred all eligible patients to the interviewers for data collection after informed consent was obtained. Trained research assistants conducted interviews after informed consent was obtained with the patients at the health care facilities, using structured questionnaires. The questionnaire was translated and back-translated by certified translators into the study languages, Khmer, Thai and Vietnamese. In each country the questionnaire was pre-tested for validity on a sample of 20 patients, which did not form part of the final sample. Interviewers were trained over three days on the interview process, ethics and the different types of TCAM. A glossary of each TCAM was provided for reference.

In Cambodia, the National Ethics Committee for Health Research of Ministry of Health in Cambodia (Reference no: 0225NECHR), in Thailand, The Committee of Research Ethics (Social Sciences) of Mahidol University (COA.No.: 2014/193.0807), and in Vietnam the The Committee of Research Ethics of Hanoi School of Public Health approved the study protocol.

### Measures

The “*International questionnaire to measure use of complementary and alternative medicine*” (I-CAM-Q) [35–37] was used. The I-CAM-Q contained three sections. Section 1 asks about “Visiting health care providers”, section 2 about the “Use of herbal medicine and dietary supplements” and section 3 about “Self-help practices”. The treatment modalities were presented in the form of a list, and respondents could provide information on their usage over the previous 12 months (yes/no) [36]. Participants were also asked to indicate whether the TCAM therapy was used for an acute illness/condition, a long-term illness, to improve general well-being, or for other reasons [36]. Moreover, respondents were asked to indicate how helpful the TCAM treatment had been [36] and from where they obtained TCAM products.

### Data analysis

Frequencies, means, and standard deviations, were calculated to describe the sample. Stepwise backward conditional logistic regression was used with the independent variables of country, gender, age, education, geolocality, and number of co-morbid medical conditions, and the dependent variable was TCAM use (provider, products and self-help) in the past 12 months. In addition, stepwise backward conditional logistic regression was used with the independent variables of individual 20 chronic conditions, and the dependent variable was TCAM use (provider, products and self-help) in the past 12 months. P levels of <0.05 was considered significant. All statistical analyses are carried out using IBM (International Business Machines Corporation) SPSS (Statistical Package for the Social Sciences) version 22.

## Results

### Sample characteristics

In Cambodia 1602 persons were approached, and all agreed to participate in the study, in Thailand 1614 were approached and 1596 (response rate 98.9 %), and in Vietnam 1601 persons were approached and the response rate was 100 %, giving a total sample of 4799. The overall mean age of participants was 50.5 years (SD = 16.3), 66.0 % were women, most (56.4 %) had Grade 6 to 12 or postsecondary education, and 55.7 % of participants resided in an urban area. Respondents had been treated in the past 12 months for hypertension (31.0 %), followed by stomach and intestinal disease (28.8 %), diabetes mellitus (13.7 %), gout and other musculoskeletal conditions (13.4 %), arthritis (12.9 %) and dyslipidaemia (8.9 %) (see Table 1); 51.6 % had two or more chronic conditions.

**Table 1:** Sample characteristics

	All	Cambodia	Thailand	Vietnam
Variable	*N* (%)	*N* (%)	*N* (%)	*N* (%)
All	4799	1602	1596	1601
Residence				
Urban	2117 (44.3)	575 (36.1)	782 (49.2)	760 (47.5)
Rural	2666 (55.7)	1017 (63.9)	809 (50.8)	840 (52.5)
Age (in years)				
18–45	1726 (37.2)	754 (47.1)	214 (13.5)	758 (52.3)
46–60	1590 (34.3)	544 (34.0)	641 (40.4)	405 (28.0)
61–101	1321 (28.5)	304 (19.0)	732 (46.1)	285 (19.7)
Gender				
Male	1611 (34.0)	357 (22.3)	646 (40.9)	608 (38.9)
Female	3131 (66.0)	1241 (77.7)	935 (59.1)	955 (61.1)
Education				
No formal education	676 (14.1)	491 (30.7)	163 (10.3)	22 (1.4)
Grade1–5	1410 (29.5)	539 (33.7)	776 (48.8)	95 (5.9)
Grade 6–12	1629 (34.0)	514 (32.1)	377 (23.7)	738 (46.2)
Postsecondary	1072 (22.4)	56 (3.5)	274 (17.2)	742 (46.5)
Religious affiliation				
None	1232 (26.0)	0 (0.0)	0 (0.0)	1232 (77.6)
Buddhist	3337 (70.3)	1522 (96.6)	1496 (94.4)	319 (20.1)
Other religion	178 (3.7)	54 (3.4)	88 (5.6)	36 (2.3)
Chronic conditions Treated in the past 12 months, for the following conditions…				
1. Hypertension	2476 (31.0)	403 (25.2)	938 (58.8)	314 (19.6)
2. Stomach and intestinal disease	2115 (28.8)	1148 (71.8)	68 (4.3)	633 (39.6)
3. Diabetes mellitus	1094 (13.7)	187 (11.7)	515 (32.3)	100 (6.2)
4. Gout and other musculoskeletal conditions, such as chronic backache	1072 (13.4)	172 (10.8)	280 (17.5)	380 (23.8)
5. Arthritis	1021 (12.9)	415 (25.9)	92 (5.8)	354 (22.1)
6. Dyslipidaemia	715 (8.9)	32 (2.0)	444 (27.9)	99 (6.2)
7. Migraine or frequent headaches	456 (6.3)	185 (11.6)	89 (5.6)	111 (6.9)
8. Cardiac failure	414 (5.2)	275 (17.2)	10 (0.6)	32 (2.0)
9. Chronic obstructive pulmonary disease (COPD)	359 (4.5)	198 (12.4)	44 (2.8)	15 (0.9)
10. Asthma	298 (3.7)	47 (2.9)	65 (4.1)	67 (4.2)
11. Kidney disease	298 (3.8)	112 (7.0)	40 (2.5)	96 (6.0)
12. Stroke	294 (3.7)	6 (0.4)	114 (7.1)	27 (1.7)
13. Liver disease	280 (3.5)	30 (1.9)	25 (1.6)	145 (9.1)
14. Coronary artery disease	209 (2.6)	31 (1.9)	53 (3.3)	23 (1.4)
15. Cardiac arrhythmias	175 (2.2)	22 (1.4)	30 (1.9)	72 (4.5)
16. Mental disorder	163 (2.3)	74 (4.6)	31 (1.9)	33 (2.1)
17. Thyroid disease	117 (1.5)	13 (0.8)	39 (2.4)	40 (2.5)
18. Parkinson’s disease	99 (1.4)	48 (3.0)	30 (1.9)	11 (0.7)
19. Cancer	93 (1.2)	7 (0.4)	19 (1.2)	46 (2.9)
20. Epilepsy	24 (0.3)	4 (0.2)	9 (0.6)	4 (0.3)
Number of chronic conditions – Mean (SD) (range 1–10)	1.9 (1.2)	2.1 (1.4)	1.8 (1.1)	1.6 (1.0)

### Health care providers consulted

Table 2 shows the participants’ utilization of various health care providers in the past 12 months. In all, 952 (26.0 %) (27.0 % in Cambodia, 26.3 % in Thailand and 23.9 % in Vietnam) had visited a TCAM provider in the past year, 19.7 % one type and 6.3 % two or more types. The most commonly consulted TCAM providers were the herbalist (17.3 %), massage therapist (6.0 %), and acupuncturist (5.5 %). Participants consulted TCAM providers mainly because of long term illness. Cambodian participants also consulted often an herbalist for acute illness. For all different types of TCAM providers more than 80 % of participants perceived the consultation as very or somewhat helpful (see Table 2).

**Table 2:** Health care providers consulted in the past 12 months

Health care providers consulted in the past 12 months	Visited	Motivation			Helpfulness
	(N, %)	Acute illness (%)	Long term illness (%)	To improve well-being or other (%)	Very/somewhat (%)
Medical practitioner					
All	4226 (90.7)	21.5	61.2	14.4	68.0/27.7
Cambodia	1449 (90.7)	32.3	58.3	23.8	53.2/45.7
Thailand	1420 (96.5)	8.4	73.9	50.9	89.3/8.5
Vietnam	1357 (85.2)	23.8	50.9	17.1	61.6/29.1
Herbalist					
All	753 (17.3)	24.7	44.4	30.8	31.8/55.3
Cambodia	325 (20.5)	46.2	28.6	25.2	14.2/73.5
Thailand	118 (9.6)	5.1	69.2	25.6	47.0/42.7
Vietnam	310 (20.0)	9.4	51.6	38.6	44.7/40.8
Massage therapist					
All	256 (6.0)	5.1	50.0	44.1	50.0/38.3
Cambodia	36 (2.3)	19.4	22.2	55.6	11.1/66.7
Thailand	188 (15.1)	3.2	59.0	32.4	58.0/33.0
Vietnam	32 (2.2)	0.0	28.1	46.9	46.9/37.5
Acupuncturist					
All	240 (5.5)	19.0	49.8	30.7	42.4/47.2
Cambodia	83 (5.2)	30.1	34.9	32.5	15.7/74.7
Thailand	43 (3.5)	4.7	67.4	27.9	81.4/16.3
Vietnam	114 (7.5)	16.2	54.3	7.6	47.6/38.1
Spiritual healer					
All	24 (0.6)	29.2	45.8	25.0	29.2/45.8
Cambodia	6 (0.4)	33.0	33.3	33.3	16.7/50.0
Thailand	3 (0.3)	0.0	100	0.0	66.7/50.0
Vietnam	15 (1.0)	33.3	33.3	33.3	26.7/46.7

### TCAM products

The use of TCAM products (herbal medicine and supplements) in the past 12 months was the most commonly used TCAM modality, with 2185 (54.8 %) (56.8 % in Cambodia, 41.9 % in Thailand and 61.9 % in Vietnam) of participants having utilized at least one type in the past 12 months, 38.5 % one type and 16.2 % two or more types. The most frequently used TCAM products were herbal medicines (41.0 %), followed by vitamins/minerals (26.5 %), and other supplements (9.7 %) in the past 12 months. The most commonly mentioned motivation for the use of TCAM products were in terms of herbal medicines and homeopathic remedies the treatment of a long term illness, and in terms of vitamins, Ginseng and other supplements to improve well-being. For the different types of TCAM products more than 80 % of participants perceived them as very or somewhat helpful (see Table 3). Examples of herbal TCAM products included: *Aloe vera, Artichoke, Artemisia vulgaris, Curcuma longa, Ganoderma lucidum, Moringa pterygosperma, Phyllanthus urinaria, Polyscias fruticose*, and *Solanum procumbens*. Herbal TCAM products were obtained in order of frequency in Cambodia from direct sale, drug store, own garden, provided by family or friends and folk remedy shop or stand, in Thailand from the folk remedy shop or stand, hospital, direct sale, provided by their family or friends and drug store, and in Vietnam from the drug store, provided by their family or friends, folk remedy shop or stand, hospital and direct sale.

**Table 3:** Use of herbal medicine and dietary supplements (TCAM products)

Variable		Motivation			Helpfulness
	Used (N, %)	Acute illness (%)	Long term illness (%)	To improve well-being (%)	Very/somewhat (%)
Herbs/herbal medicine					
All	1845 (41.0)	14.3	50.8	39.4	47.9/41.7
Cambodia	708 (44.5)	23.9	55.6	20.5	35.6/54.6
Thailand	473 (34.6)	6.1	50.4	43.4	57.3/33.0
Vietnam	664 (42.9)	9.9	45.8	44.3	54.3/34.2
Vitamins/minerals					
All	1180 (26.5)	7.1	23.0	70.0	58.8/32.5
Cambodia	358 (22.6)	10.9	10.9	78.1	64.1/31.4
Thailand	242 (18.5)	7.9	21.2	70.9	64.5/31.0
Vietnam	580 (37.3)	4.3	31.3	64.3	53.0/33.9
Homeopathic remedies					
All	86 (2.0)	8.4	73.5	18.0	78.3/10.8
Cambodia	54 (3.4)	1.9	94.3	3.8	88.7/9.4
Thailand	11 (0.9)	9.1	45.5	45.5	81.8/9.1
Vietnam	21 (1.4)	26.3	31.6	42.1	47.4/15.8
Ginseng					
All	156 (3.6)	2.0	12.5	85.6	50.7/32.9
Cambodia	1 (0.1)	0.0	0.0	100	0.0/100
Thailand	65 (5.2)	1.6	18.8	79.7	59.4/28.1
Vietnam	90 (6.0)	2.3	8.0	89.6	44.8/35.6
Other supplements					
All	396 (9.7)	4.8	25.3	75.3	50.5/35.0
Cambodia	12 (0.8)	8.3	8.3	83.3	16.7/50.0
Thailand	150 (14.5)	2.0	24.8	73.2	52.0/44.7
Vietnam	234 (15.5)	6.5	26.4	67.0	51.3/28.8

### Self-help TCAM

In terms of self-help TCAM, 1714 (40.9 %) (50.7 % in Cambodia, 48.1 % in Thailand and 25.4 % in Vietnam) had used self-help TCAM in the past 12 months, the most common being prayer for own health (30.1 %), meditation (13.9 %) and relaxation techniques (9.9 %). The most commonly mentioned motivation for the use of self-help TCAM such as meditation and prayer for own health was to improve well-being. The rating of TCAM self-help practices as very or somewhat helpful was the highest for meditation 92 %, followed by yoga 82 %, relaxation techniques 75 %, prayer 71 %, and visualization 47 % (see Table 4). The prevalence of TCAM provider and/or TCAM products use was 59.6 %) (60.5 % in Cambodia, 47.6 % in Thailand and 66.8 % in Vietnam), and the prevalence of any TCAM use (providers, products or self-care) was 76.7 % (82.2 % in Cambodia, 69.0 % in Thailand and 77.0 % in Vietnam).

**Table 4:** TCAM self-help practices

Variable		Motivation			Helpfulness
	Used (N, %)	Acute illness (%)	Long term illness (%)	To improve well-being (%)	Very/somewhat (%)
Prayer for own health					
All	1346 (30.1)	6.5	12.1	81.4	35.5/35.3
Cambodia	571 (35.9)	13.9	7.7	79.4	1.9/48.9
Thailand	523 (40.0)	0.6	12.3	87.2	78.2/18.0
Vietnam	352 (16.1)	2.0	21.8	76.1	23.0/40.7
Meditation					
All	619 (13.9)	2.8	13.8	83.5	61.1/31.1
Cambodia	145 (9.1)	2.8	4.8	92.4	23.4/70.3
Thailand	383 (29.0)	2.4	16.3	81.5	78.5/17.8
Vietnam	91 (5.9)	4.4	17.8	77.7	47.8/24.4
Relaxation techniques					
All	475 (10.9)	2.1	11.6	86.2	33.8/41.9
Cambodia	287 (18.1)	1.7	3.1	95.1	19.6/47.9
Thailand	100 (8.1)	0.0	23.0	77.0	69.0/26.0
Vietnam	88 (5.7)	5.7	26.4	75.7	40.2/40.2
Visualization					
All	421 (9.8)	1.0	2.4	96.7	5.7/41.3
Cambodia	402 (25.6)	0.7	1.5	78.8	5.2/41.0
Thailand	3 (0.3)	0.0	66.7	33.3	33.3/66.7
Vietnam	16 (1.1)	6.2	12.5	43.8	12.5/43.1
Yoga					
All	233 (5.4)	1.3	12.1	86.6	41.8/40.9
Cambodia	97 (6.1)	3.1	5.2	91.7	7.2/69.1
Thailand	69 (5.6)	0.0	2.9	97.1	73.5/16.2
Vietnam	67 (4.4)	0.0	31.3	68.6	59.7/25.4

### Sources of influence

Health personnel in the health care facility (27.9 %) were the main source of influence or awareness of TCAM on chronic disease patients in relation to TCAM use, followed by family members (21.2 %), friends (16.1 %), health personnel outside of the health care setting (15.6 %), mass media (9.6 %), other patients (8.4 %), migrant advertisers (7.2 %), TCAM practitioner (4.4 %), religious institution (0.6 %) and other (8.4 %).

### Associations with any TCAM use

In multivariate logistic regression analyses, older age, rural residence and having two or more chronic conditions was associated with the use a TCAM provider; being female, urban residence, residing in Vietnam and having two or more chronic conditions was associated with the use of TCAM products; and being female, older age, rural residence, higher formal education, and residing in Cambodia was associated with the use of TCAM self-help practices (see Table 5). Further, based on multivariate logistic regression analyses, having the following chronic conditions (gout, arthritis, migraine, cardiac failure, kidney disease, stroke and mental disorder) were associated with the utilization of TCAM providers, having stomach and intestinal diseases, gout, arthritis, stroke, coronary artery disease, cardiac arrthythmias and cancer were associated with the use of TCAM products, and having diabetes, arthritis, dyslipidaemia, migraine and thyroid disease were associated with the use if TCAM self-help practices (see Table 6).

**Table 5:** Associations with different types of TCAM use

Variable	TCAM provider	TCAM products	TCAM self-help
	Adjusted Odds Ratio (95 % CI)^a,b^	Adjusted Odds Ratio (95 % CI)^a,c^	Adjusted Odds Ratio (95 % CI)^a,d^
Sex			
Female		1.00	1.00
Male		0.70 (0.61–0.81)***	0.57 (0.49–0.66)***
Age (in years)			
18–45			1.00
46–60			1.43 (1.21–1.69)***
61–101	1.19 (1.02–1.40)*		2.11 (1.75–2.55)***
Geolocality			
Rural	1.00	1.00	1.00
Urban	0.54 (0.46–0.64)***	1.30 (1.14–1.49)***	0.53 0.46–0.60)***
Education			
No formal education			1.00
Grade1–5			1.30 (1.05–1.60)*
Grade 6–12			1.28 (1.02–1.59)*
Postsecondary			2.07 (1.57–2.73)***
Country			
Cambodia		1.00	1.00
Thailand		0.57 (0.48–0.67)***	0.75 (0.63–0.90)**
Vietnam		1.54 (1.31–1.81)***	0.31 (0.25–0.38)***
Chronic conditions			
One	1.00	1.00	1.00
Two	1.26 (1.05–1.51)*	1.36 (1.16–1.59)***	–
Three or more	2.16 (1.74–2.55)***	1.63 (1.37–1.95)***	1.14 (0.99–1.32)

^a^ Backward stepwise conditional LR selection of variables

^b^ For Hosmer and Lemeshow Chi-square 4.26, df 8, 0.833; Cox and Snell R^2^ 0.04; Nagelkerke R^2^ 0.06

^c^ For Hosmer and Lemeshow Chi-square 33.40, df 8, 0.001; Cox and Snell R^2^ 0.05; Nagelkerke R^2^ 0.06

^d^ For Hosmer and Lemeshow Chi-square 19.50, df 8, 0.012; Cox and Snell R^2^ 0.10; Nagelkerke R^2^ 0.12

***P < 0.001; **P < 0.01; *P < 0.05

**Table 6:** Associations between chronic conditions and different types of TCAM use

Chronic conditions treated in the past 12 months, for the following conditions…	TCAM provider	TCAM products	TCAM self-help
	Adjusted Odds Ratio	Adjusted Odds Ratio	Adjusted Odds Ratio
1) Hypertension		0.85 (0.74–0.99)*	
2) Stomach and intestinal disease		1.91 (1.16–2.19)***	0.54 (0.42–0.70)***
3) Diabetes mellitus			1.48 (1.05–2.08)*
4) Gout and other musculoskeletal conditions, such as chronic backache	1.94 (1.59–2.37)***	1.58 (1.32–1.88)***	0.33 (0.23–0.48)***
5) Arthritis	2.01 (1.66–2.44)***	1.26 (1.06–1.50)***	2.04 (1.58–2.65)***
6) Dyslipidaemia			5.68 (1.87–17.31)**
7) Migraine or frequent headaches	1.69 (1.29–2.20)***		1.72 (1.21–2.45)**
8) Cardiac failure	1.34 (1.05–1.81)*	0.79 (0.61–1.01)	
9) Chronic obstructive pulmonary disease (COPD)			0.63 (0.45–0.89)**
10) Asthma			
11) Kidney disease	1.42 (1.03–1.95)*		
12) Stroke	4.31 (2.99–6.22)***	2.11 (1.45–3.07)***	
13) Liver disease			
14) Coronary artery disease	1.54 (0.96–2.46)	1.70 (1.06–2.70)*	0.47 (0.20–1.09)
15) Cardiac arrhythmias		2.28 (1.42–3.64)***	
16) Mental disorder	2.12 (1.42–3.19)***		
17) Thyroid disease			7.78 (1.52–39.91)*
18) Parkinson’s disease			
19) Cancer		1.89 (1.12–3.19)*	0.23 (0.04–1.45)
20) Epilepsy		0.31 (0.10–0.99)*	

***P < 0.001; **P < 0.01; *P < 0.05

## Discussion

This study found a high prevalence of any TCAM use (76.7 %) (providers = 26.0 %, products = 54.8 % and self-care 54.8 %) among this sample of chronic disease patients in Cambodia, Thailand and Vietnam. These findings compare with previous surveys among chronic disease patients in Malaysia (63.9 %) [28], Singapore (22.7 %) [27] and medical patients in Thailand (52.5 %) [30]. Among the three study countries, the utilization of TCAM providers were similar, while the use of TCAM products was highest in Vietnam and lowest in Thailand, and the use of self-help TCAM was highest in Cambodia and lowest in Vietnam. The latter finding may be attributed to the low rate of “prayer for own health” in Vietnam, having a large population indicating having no religion.

The study found a wide range of different TCAM practices, ranging from TCAM providers of the herbalist, massage therapist, and acupuncturist, the TCAM products herbal medicines, vitamins/minerals, and other supplements and TCAM self-help practices including prayer for own health, meditation and relaxation techniques. This finding has been confirmed in previous studies in Vietnam, e.g., chiropractic, diet supplements, and dietary therapy [37]. Main reasons for consulting TCAM providers were because of long term illness; Cambodian participants also consulted often an herbalist for acute illness. The latter finding may be because of lack of access to conventional health care. The use of TCAM products (herbal medicines and homeopathic remedies) were mainly used for treatment of a long term illness, and in terms of vitamins, Ginseng and other supplements, and the use of self-help TCAM such as meditation and prayer for own health were mostly used to improve well-being.

The higher usage of TCAM among older patients found in this study compares with previous studies [28]. As, also found in some other studies [31, 32], female gender was in this study associated with the use of TCAM products and self-help TCAM. However, there were no gender differences in terms of the use of TCAM providers, as also found in a Malaysian study [28]. This study found that TCAM self-help practices increased with higher educational levels of the chronic disease patients, as also found in some previous studies [28, 31, 32], while educational levels were not associated with the use of TCAM providers and TCAM products. One possible explanation, according to Hasan et al. [28], of TCAM self-help use among educated patients may be that they are more aware about TCAM self-help. In terms of geolocality, in this study chronic disease patients residing in rural areas were more likely to use a TCAM provider and TCAM self-help practices than people residing in urban areas; and the reverse was true for the use of TCAM products. The latter finding was mainly due to a more prevalent use of vitamins and minerals in chronic disease patients in urban than rural areas. The higher use of vitamins and minerals in urban areas may be attributed to greater awareness of the need to use them. On the other hand, the greater use of TCAM providers and self-help in rural than urban areas may be attributed to poorer access to and poorer quality of conventional health care services in rural areas [37].

This study confirms previous research indicating that TCAM users were more likely to suffer from two or more chronic conditions [16, 18, 19, 21, 27, 33]. Further, we found in agreement with previous studies (e.g.,[27] that patients with specific chronic conditions such as stroke, arthritis or musculoskeletal conditions were more likely to use TCAM [27, 30, 38]. The association between having a mental disorder and TCAM use has also been found among U.S. Cambodian refugees [39]. Persons with diabetes in this study were more likely to use TCAM self-help practices such as prayer for own health. Similarly, in a national US study [40] found that persons with diabetes were more likely to use prayer, but less likely to use herbs, yoga, or vitamins than people without diabetes. Regarding the association between cancer and the use of TCAM products, in a number of studies reviewed by Bishop and Lewith [17] such association was found.

Similar to this study, Hasan et al. [28] found among chronic disease patients in Malaysia that the majority felt that TCAM had improved their health condition. This finding seems to underline the beneficial role of TCAM in this study population. Further, this study found that the three main sources of influence or awareness of TCAM were health care professionals, family members and friends. A similar result was found among chronic disease patients in Malaysia [28]. It is possible that health care providers advise on the use of TCAM such as vitamins and other supplements as a complementary rather than alternative treatment [28]. Satyapan et al. [21] found among hospital patients in Bangkok that more than 60 % had received knowledge of herbal drugs from mass media.

### Study limitations

While the study was conducted in specific geographic areas in the study countries, findings cannot be generalized to other areas in study countries. There may have been a recall bias resulting in an underestimation of TCAM use, given that study participants were retrospectively asked over the past 12 months about TCAM utilization. Further, interviewers may have influenced some of the responses of the participants due to the way they could have explained difficult TCAM modalities such as homeopathy and visualization. There could have been misinterpretation of herbal and homeopathic remedies, as found in some previous study [36]. Another limitation was that the medical provider-patient communication about TCAM [28] use was not assessed, and should be included in future studies.

## Conclusions

A large proportion of patients with over 20 chronic diseases reporting to health care facilities in Cambodia, Thailand and Vietnam were using TCAM and were satisfied with its use. The use of TCAM in this population may be beneficial, especially since some patients may have limited access to conventional health care facilities. It is important that medical health care providers increase their awareness and understanding of potential TCAM use by their chronic disease patients, especially regarding potential interactions of specific herbal remedies with conventional medicines.

## Abbreviations

ASEAN: Association of Southeast Asian Nations
COPD: chronic obstructive pulmonary disease
TCAM: traditional, complementary and alternative medicine
